# Efficacy of Tacrolimus Versus Clobetasol in the Treatment of Vitiligo

**DOI:** 10.7759/cureus.11985

**Published:** 2020-12-08

**Authors:** Hassan Mumtaz, Salwa Anis, Ambreen Akhtar, Masooma Rubab, Ayesha Zafar, Nayab Niazi, Hina Bahadur, Abdul Subhan Talpur, Muhammad A Shafiq, Tehreem Fatima

**Affiliations:** 1 Urology, Guy's and St Thomas' Hospital, London, GBR; 2 General Medicine, Surrey Docks Health Center, London, GBR; 3 Surgery, KRL Hospital, Islamabad, PAK; 4 Internal Medicine, Ayub Teaching Hospital, Abbottabad, PAK; 5 Internal, Holy Family Hospital, Islamabad, PAK; 6 Medicine, Combined Military Hospital, Multan, USA; 7 Physiology, HITEC University, Rawalpindi, PAK; 8 Medical Department, Doctor Akbar Niazi Teaching Hospital, Islamabad, PAK; 9 Medical Unit, Mayo Hospital Lahore, Lahore, PAK; 10 Medicine, Liaquat University of Medical and Health Sciences, Jamshoro, PAK; 11 Internal Medicine, California Institute of Behavioral Neurosciences & Psychology, Fairfield, USA; 12 Internal Medicine, Rawalpindi Medical University, Islamabad, PAK

**Keywords:** vitiligo, tacrolimus, clobetasol propionate, topical steroids, topical immunomodulators

## Abstract

Introduction

Vitiligo is an acquired pigmentary disorder of the skin and mucous membranes which is characterized by circumscribed depigmented macules and patches. Vitiligo is a progressive disorder in which some or all of the melanocytes in the affected skin are selectively destroyed. Around 0.5-2% of the world population is affected by vitiligo and the average age of onset is 20 years. The objective of the study was to evaluate the efficacy of tacrolimus versus clobetasol in the treatment of vitiligo. It is an open randomized control trial conducted in the Department of Dermatology, Nishtar Hospital, Multan for six months.

Methods

One hundred sixty-two patients of vitiligo were included in the study. The disease was diagnosed on basis of clinical features and the Standard Assessment scale proposed by Hossain which was used to monitor and grade the response. Patients were randomly allocated into two groups by lottery method having 81 patients in each group. Group A was given tacrolimus whereas Group B was given clobetasol. Patients were followed up every four weeks. On the 12^th^ week of treatment, effectiveness was assessed by measuring the Assessment scale proposed by Hossain. The results of the two groups were then compared.

Results

Sixty-three patients (38.9%) were males whereas 99 patients (61.1%) were females. The mean age of the patients included in the study was 29.68 + 8.162 years. The mean weight of the patients was 62.25 + 9.529 Kg. Out of 162, treatment was effective in 89 patients (54.9%) whereas in 73 patients (45.1%) the treatment was ineffective. In Group A (tacrolimus), 42 patients (51.9%) had effective treatment (on the complete resolution of symptoms) whereas 39 patients (48.1 %) had ineffective treatment. In Group B (clobetasol), 47 patients (58%) had effective treatment, and the rest (34, 42%) had ineffective treatment. A Chi-Square test was applied to compare the efficacy of the two groups. There was no statistically significant difference in both the groups in terms of efficacy. Group B was numerically superior in terms of effective treatment (47 versus 42) but not superior statistically.

Conclusion

Comparison of tacrolimus and clobetasol in patients of vitiligo showed no significant difference in the efficacy of the two groups. It can be concluded that tacrolimus may be considered superior to corticosteroids as its local and systemic adverse effects are less.

## Introduction

Vitiligo is an acquired pigmentary disorder of the skin, characterized by the loss of function of melanocytes in the epidermis and well-circumscribed, asymptomatic pearly white macules varying in size and shape which tend to extend and increase centrifugally with time in an unpredictable way [1].

Two of the major theories of the pathogenesis of vitiligo are the autoimmune theory and the autocytotoxicity theory [2]. The former autoimmune theory speculates that patients with vitiligo form autoantibodies against melanocytes. The latter autocytotoxicity theory postulates that melanocytes are destroyed either by themselves through self-generation of melanin precursors (or metabolites) or by keratinocytes, which release chemicals that generate oxidative stresses.

Current treatment options are far from ideal and there is still a search for a treatment that would give consistent, safe, and long-term cure by repigmentation. Topical corticosteroids e.g. 0.05% clobetasol propionate, 0.05% betamethasone dipropionate, 0.005% fluticasone propionate are indicated for the treatment of limited areas of vitiligo. Various studies have shown that as many as 57% to 64% of patients respond at least partially to the application of potent topical corticosteroids, e.g. 0.05% clobetasol propionate [3], the risk of cutaneous atrophy and telangiectasias, especially on the face and in intertriginous areas, and of ocular adverse events when applied to periorbital sites, precludes the prolonged use of topical corticosteroids.

Considering the autoimmune hypothesis of vitiligo pathogenesis due to humoral and cellular dysfunction, the use of calcineurin inhibitors tacrolimus and pimecrolimus for the treatment of vitiligo seems reasonable. Tacrolimus works on the immune system by its ability to inhibit T lymphocyte activation and directly on skin cells. Recently, reports of successful monotherapy with tacrolimus in the treatment of vitiligo have appeared and certain comparative studies against potent topical corticosteroids, psoralen-Ultraviolet A (PUVA) and narrowband ultraviolet B (UVB) as well as in non-comparative studies have shown incomplete cure with some re-pigmentation of the lesions in 73%-83.3% of the patients [4].

Tacrolimus is a new treatment option for vitiligo and it does not cause corticosteroid-related side effects such as atrophy, telangiectasia, or adverse potential ocular effects of topical corticosteroids. This data has been collected from studies that have been conducted internationally but local data is not available in reference to the use of these drugs in Pakistan.

## Materials and methods

This randomized control trial was done in the Department of Dermatology, Nishtar Hospital, Multan. The time duration of the study was six months. The sample size was taken as 162 with 81 patients in each group. The sampling technique used was nonprobability consecutive sampling.

Patients of both genders aged between 15 to 40 years having vitiligo for at least the last three months duration were included in the study. Pregnant or lactating females and women with childbearing potential not using an adequate contraception method were excluded from the study. Patients with a known sensitivity to the study drug or class of study drug and suffering from co-morbid conditions like neurological or psychiatric disorders, an autoimmune disease (especially thyroid disease), immune defects, heart deficiency, kidney failure, or previous or current history of neoplasms were also excluded from the study.

Data collection procedure

One hundred sixty-two patients with vitiligo were included in the study. The disease was diagnosed on basis of clinical features and the Assessment scale proposed by Hossain was used to monitor and grade the response. Patients were randomly allocated into two groups by the lottery method. Group A, having 81 patients were given topical 0.1% tacrolimus ointment whereas Group B, having 81 patients were given topical 0.05% clobetasol propionate ointment. Patients were followed up every four weeks. On the 12th week of treatment, effectiveness was assessed by measuring the Assessment scale proposed by Hossain.

Data analysis was done using Statistical Package for Social Sciences (SPSS) version 21 (IBM Corp., Armonk, NY, USA). Descriptive statistics were used to calculate mean and standard deviation (SD) for age and weight. Frequencies and percentages were presented for gender and effectiveness of drugs in both groups. A Chi-Square test was applied to compare the effectiveness between the two groups. P-value of < 0.05 was taken as significant. Stratification of age, weight, and gender was done to control the effect modifiers and the Chi-Square test was applied to see the effect of these on outcome variables.

## Results

A total of 162 patients were inducted into the study. 63 patients (38.9%) were males whereas 99 patients (61.1%) were females. The mean age of the patients included in the study was 29.68 + 8.162 years. The mean age of males was 29.22 + 6.812 years and that of females was 29.97+ 8.937 years.

The mean age in Group A (tacrolimus) was 30.12+ 7.264 years. The mean age of males in this group was 30.15 + 6.062 years. Females had a mean age of 30.11 + 7.849 years. The mean age in Group B (clobetasol) was 29.23+ 8.995 years. The mean age of males in this group was 28.53 + 7.331 years. Females had a mean age of 29.80 + 10.179 years (Table 1).

**Table 1:** Age in Years of Both Groups

Medicine	Sex	Mean	Std. Deviation
0.05% clobetasol propionate (Group B)	Male	28.53	7.331
	female	29.80	10.179
	Total	29.23	8.995
0.1% tacrolimus (Group A)	Male	30.15	6.062
	Female	30.11	7.849
	Total	30.12	7.264

The mean weight of the patients was 62.25 + 9.529 kg. The mean weight of the male population was 70.13 + 6.966 kg. The mean weight of the females was 57.23 + 7.291 kg. The mean weight of patients in Group A (tacrolimus) was 62.77 + 9.023 kg. The mean weight of males in the group was 71.93 + 5.61 kg while the females had a mean weight of 58.19 + 6.428 kg (Table 2). The mean weight of patients in Group B (clobetasol) was 61.73 + 10.04 kg. Males had a mean weight of 68.78 + 7.43 kg while the females had a mean weight of 56.09 + 8.135 kg as demonstrated in Table 2. 

**Table 2:** Weight in Kilograms of Both Groups

Medicine	Sex	Mean	Std. Deviation
0.05% clobetasol propionate (Group B)	Male	68.78	7.430
	female	56.09	8.135
	Total	61.73	10.040
0.1% tacrolimus (Group A)	Male	71.93	5.961
	female	58.19	6.428
	Total	62.77	9.023

Out of 162, treatment was effective in 89 patients (54.9%) whereas in 73 patients (45.1%) the treatment was ineffective.

**Figure 1:**
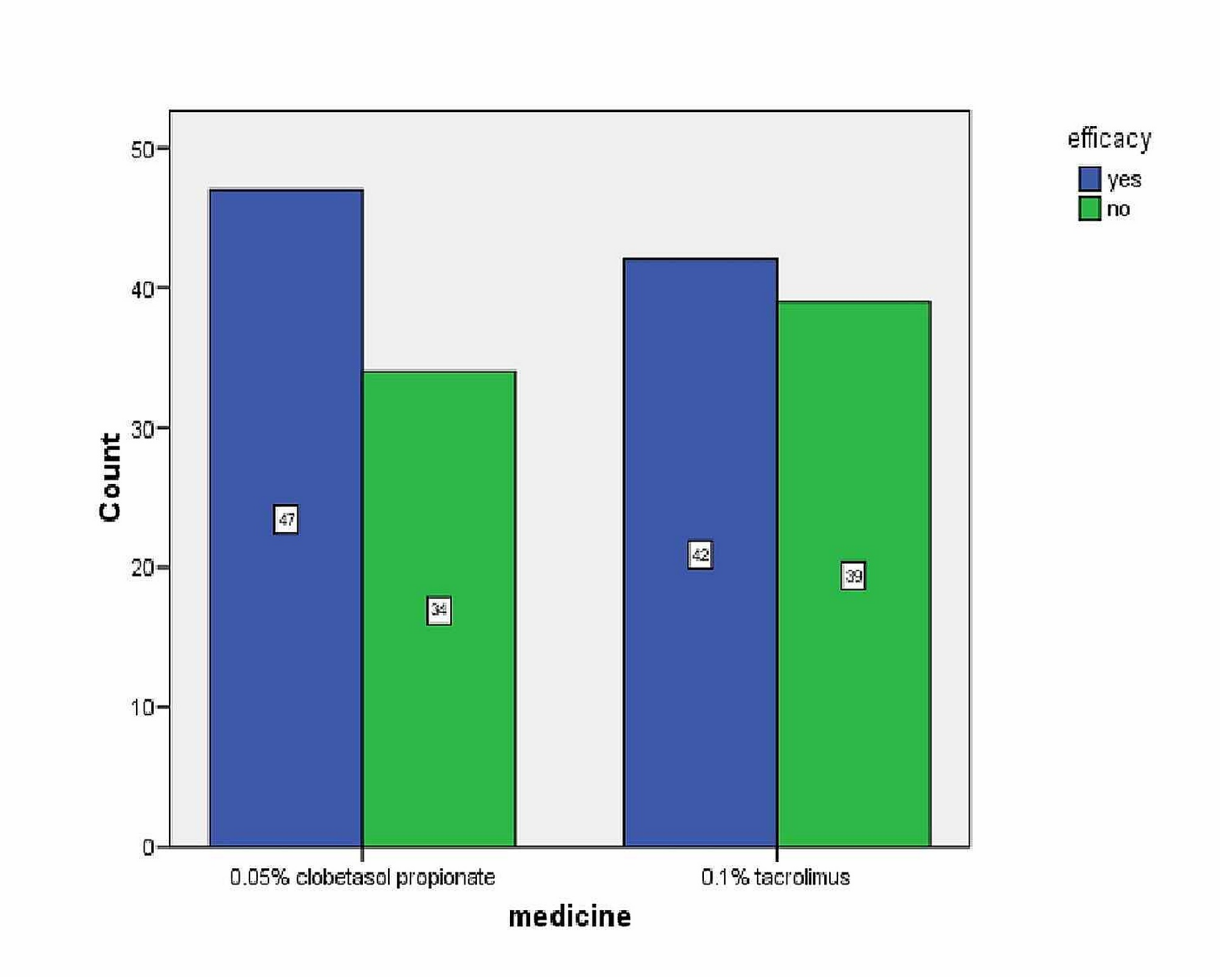
Efficacy of Both Drugs

In Group A (tacrolimus), 42 patients (51.9%) had effective treatment whereas 39 patients (48.1 %) had ineffective treatment. In Group B (clobetasol), 47 patients (58%) had effective treatment, and the rest 34 patients (42%) had ineffective treatment (Table 3).

**Table 3:** Efficacy of Group A vs Group B

Medicine			Frequency	Percent
0.05% clobetasol propionate (Group B)	Effective	yes	47	58.0
		no	34	42.0
		Total	81	100.0
0.1% tacrolimus (Group A)	Effective	yes	42	51.9
		no	39	48.1
		Total	81	100.0

In males, 33 patients (52.4%) had effective treatment whereas 30 males (47.6 %) had ineffective treatment. Similarly, 56 females (56.6%) had effective treatment (Table 4).

**Table 4:** Efficacy of Both Drugs With Respect to Gender

Gender			Frequency	Percent
Male	Effective	Yes	33	52.4
		No	30	47.6
		Total	63	100.0
Female	Effective	Yes	56	56.6
		No	43	43.4
		Total	99	100.0

There was no statistically significant difference in both the groups in terms of efficacy. Group B was numerically superior in terms of effective treatment (47 versus 42) but not superior statistically.

There was no statistically significant difference in efficacy when correlated with age (p-value = 0.469), weight (p-value = 0.974), and gender (p-value = 0.604) in both the groups.

## Discussion

Vitiligo is the most commonly occurring pigmentation disorder which affects both sexes equally [5]. It is an autoimmune disease in which autoantibodies are produced against the individual’s own melanocytes leading to depigmentation of skin and overlying hair. Its pathogenesis is still not completely understood but studies have shown that both genetic factors as well as environmental triggers play a complex role [6].

Vitiligo is characterized by the presence of depigmented patches which are usually multiple, present bilaterally, variable in size, and tend to increase progressively with time [5].

Various treatment modalities, including medical and surgical, have been studied so far for the treatment of vitiligo [7]. One of the most commonly used treatment options is the use of potent topical corticosteroids. They are effective in this and other autoimmune diseases due to their immunosuppressive effects. Of these, 0.05% clobetasol propionate has been used frequently due to its high potency [8]. The drawback in treating patients with long-term potent topical corticosteroids is their adverse effects which may lead to thinning of skin, striae, telangiectasias, acne, rosacea, and atrophy of skin [9].

Another option that is being increasingly used in the past years for the treatment of vitiligo patients is topical calcineurin inhibitors like tacrolimus and pimecrolimus which decrease the responsiveness of T lymphocytes to foreign antigens by inhibiting the transcription of genes involved in the activation of inflammatory mediators. One of the main benefits of using long-term topical tacrolimus is that it does not lead to the adverse effects caused by prolonged topical corticosteroid use. They may lead to only mild itching or burning sensation at the site of application.

Various studies have been conducted internationally to evaluate the efficacy of topical corticosteroids and topical calcineurin inhibitors in treating patients with vitiligo. A study conducted in 2009 showed that treatment of vitiligo with tacrolimus ointment for a period of four months resulted in some repigmentation in about 83.3% of the patients [4]. Another study conducted in 2008 compared the results of topical immunomodulators and topical corticosteroids and showed that repigmentation was seen earlier in patients treated with topical immunomodulators [3]. Another study conducted in 2003 compared clobetasol propionate ointment and topical tacrolimus ointment. The results showed some repigmentation in about 90% of the patients with not much significant difference between the two groups. The study concluded that since repigmentation rates with both drugs were about the same but more adverse effects were seen with corticosteroids, topical tacrolimus may be preferred due to its fewer side effects [10].

We compared topical 0.05% clobetasol propionate ointment and 0.1% tacrolimus ointment in patients with vitiligo. Repigmentation was measured by monitoring the size and color of the lesions along with the presence or absence of follicular repigmentation. It was seen that partial repigmentation was seen in 51.9% of the patients treated with tacrolimus and 58% of the patients treated with clobetasol propionate. There was no significant statistical difference seen between the efficacy of these two drugs.

## Conclusions

Comparison between the effectiveness of topical 0.05% clobetasol propionate and 0.1% tacrolimus ointment showed partial repigmentation rates in patients. The results were comparable with no significant statistical difference between the two groups. Tacrolimus may therefore be preferred due to its fewer side effects but further studies are required to monitor them.
